# Multi Step Selection in Ig H Chains is Initially Focused on CDR3 and Then on Other CDR Regions

**DOI:** 10.3389/fimmu.2013.00274

**Published:** 2013-09-17

**Authors:** Gilad Liberman, Jennifer Benichou, Lea Tsaban, Jacob Glanville, Yoram Louzoun

**Affiliations:** ^1^Gonda Multidisciplinary Brain Research Center, Bar-Ilan University, Ramat Gan, Israel; ^2^Department of Mathematics, Bar-Ilan University, Ramat Gan, Israel; ^3^Protein Engineering and Applied Quantitative Genotherapeutics, Rinat-Pfizer Inc., South San Francisco, CA, USA

**Keywords:** adaptive evolution, phylogenetic tree, immune system, micro-evolution, tree shapes

## Abstract

Affinity maturation occurs through two selection processes: the choice of appropriate clones (clonal selection), and the internal evolution within clones, induced by somatic hyper-mutations, where high affinity mutants are selected for. When a final population of immunoglobulin sequences is observed, the genetic composition of this population is affected by a combination of these two processes. Different immune induced diseases can result from the failure of regulation of clonal selection or of the regulation of the within clone affinity maturation. In order to understand each of these processes separately, we propose a mixed lineage tree/sequence based method to detect within clone selection as defined by the effect of mutations on the average number of offspring. Specifically, we measure the imbalance in the number of leaves in lineage trees branches following synonymous and non-synonymous (NS) mutations. If a mutation is positively selected, we expect the number of leaves in the sub-tree below this mutation to be larger than in the parallel sub-tree without the mutation. The ratio between the number of leaves in such branches following NS mutations can be used to measure selection within a clone. We apply this method to the sampled Ig repertoire from multiple healthy volunteers and show that within clone selection is positive in the CDR2 region and either positive or negative in the CDR3 and FWR3 regions. Selection occurs already at the IgM isotype level mainly in the DH gene region, with a strong negative selection in the join region. This is followed in the later memory stages in the CDR2 region. We have not studied here the FWR1 and CDR1 regions. An important advantage of this method is that it is very weakly affected by the baseline mutation model or by sampling biases, as are most synonymous to NS mutations ratio based methods.

## Introduction

The humoral adaptive immune response is based on the production of high affinity antibodies against pathogenic antigens. These antibodies are produced through an affinity maturation process, where high affinity antibodies are produced from a large number of cells with different B Cell Receptors (BCRs). The affinity maturation process involves two stages. The first one is clonal selection, where a set of cells with initially mid-high affinity of their BCR to the antigen are expanded. Following this stage, each clone passes a within clone increase in affinity, through somatic hypermutation (SHM) (1) that alter the properties of the BCR, mainly in the complementarity determining region (CDR)3 region (2). The end product of this affinity maturation process is a large population of B cells, with varying BCR that often contain at least one high affinity clone (3).

While the choice of a clone is equivalent to the selection of one specie among many, the dynamics of specific clones in the B cell response against pathogens (4, 5) is a classical example of a process involving rapid asexual reproduction, where constant diversification and adaptation occurs following a high mutation rate. While many tools to study the evolution of species have been developed, tools for the analysis of within population evolution are lacking. We here propose a new method to analyze the within clone affinity maturation process and use it to analyze the healthy B cell repertoire in a large cohort of healthy volunteers.

Multiple methods have been proposed for the measures of selection in populations or between populations, in the sense of evolving toward a higher fitness phenotype (Table 1). A now classical measure is the synonymous (S) to non-synonymous (NS) mutations. Specifically, a comparison of the observed and expected NS/(NS + S) ratios is often used as a measure for selection. The expected ratio is calculated based on an underlying mutation probability model [e.g., Ref. (6–8)], or based on genetic regions where no selection is assumed to occur (9). An increased frequency of NS mutations is treated as an indication for positive selection and a decreased one indicates negative selection. Important drawbacks of such methods are: (a) their strong sensitivity to the baseline mutation model (i.e., the expected probability of each mutation type), especially when the mutations rate is position dependent, as happens for example in immunoglobulin sequences (10), and (b) the effect of sampling biases. However, the main problem lies in the fact that they were developed for an analysis of the comparison between species (clones in this case), and not within a specie.

**Table 1:** **List of existing methods based on their reference (first column), the method they use (second column), whether they can detect the direction of selection (third column) and the baseline to which they compare in order to define if selection took place (last column)**.

Reference	Method	Directional	Baseline reference
Nei and Gojobori (6)	S vs. NS ratio	Yes	Mutation probability model
Yang (7)	S vs. NS ratio	Yes	Mutation probability model
Yang and Nielsen (8)	S vs. NS ratio	Yes	Mutation probability model
Shlomchik et al. (9)	S vs. NS ratio	Yes	Genetic region with no selection pressure
Hershberg et al. (10)	S vs. NS ratio	Yes	Position dependent mutation probability model
Sackin (11)	Tree morphology	No	Yule model
Colless (12)	Tree morphology	No	Yule model
Tajima (16), others	Mutation statistic	No	Naive evolution

A different approach for detecting selection is to use properties of lineage trees. Two of the most powerful such methods are Sackin’s and Colless’s statistics (11–14). Sackin’s index is the average root’-leaf distance (over all leaves). Colless’s index is the sum of imbalance over all nodes, where a node’s imbalance is taken to be the difference in number of leaves between the bigger and smaller sub-trees. These measures are tested vs. a neutral model, which is usually the Yule model, where a tree is constructed by giving each branch the same probability to split (15). Other statistics do not use trees but are based on properties of the full sequences, most notably Tajima’s D (16). Such methods have two well-known limitations. They do not distinguish between S and NS mutations and statistical power is lost. However, perhaps the most significant limitation is that in most cases, these methods cannot differ between different types of selection, e.g., positive and negative ones.

We here offer a more direct approach to measure selection within a clone, as well as a better definition of its meaning. This new method overcomes limitations of the S to NS mutation ratio and of the tree shape based selection detection methods, by accounting for the completing information found in each of the two, that is, the classification into mutation types, and the imbalance between different sub-trees.

## Materials and Methods

### Selection score

**Figure 1:**
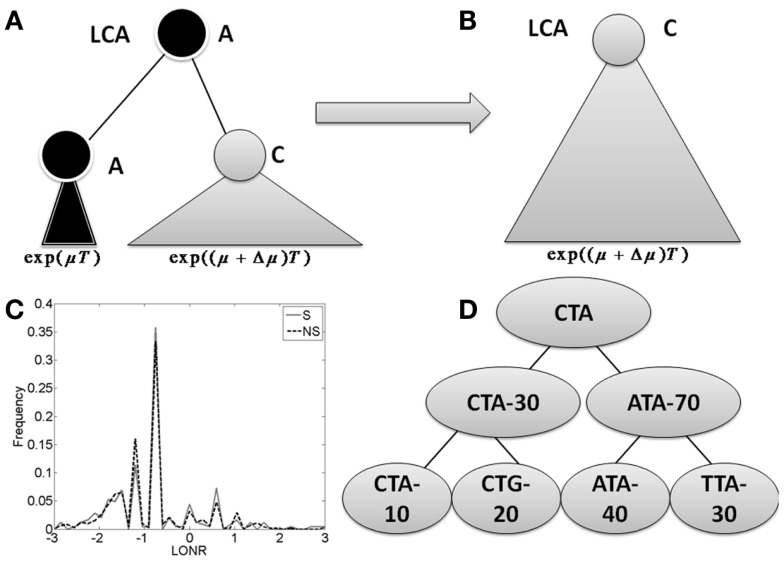
**The branch imbalance framework and examples**. **(A)** Schematic view of a branch corresponding to a mutation event. Following a mutation, the population can be expanded (or reduced), the advantage will lead to an exponentially growing difference in the number of offspring in parallel branches descending from the same internal origin. *T* is the time from the mutation to the sampling time. **(B)** After some time, one branch will take over the entire sample, and the information carried in the ratio between the branches will be lost. **(C)** LONR values histogram for one simulated sequence pool, simulated under naive multiplication from unique ancestral sequence. While the average is not 0, there is no difference between branches following S and NS mutations. **(D)** Example of tree. In the left branch, a mutation occurred from CTA to CTG, and the ratio between the mutated and un-mutated branches number of offspring is 20/10. In the right branch, a mutation from ATA to TTA occurred, with a ratio of 30/40. In the root, a mutation from CTA to ATA occurred with a ratio of 70/30.

**Figure A1:**
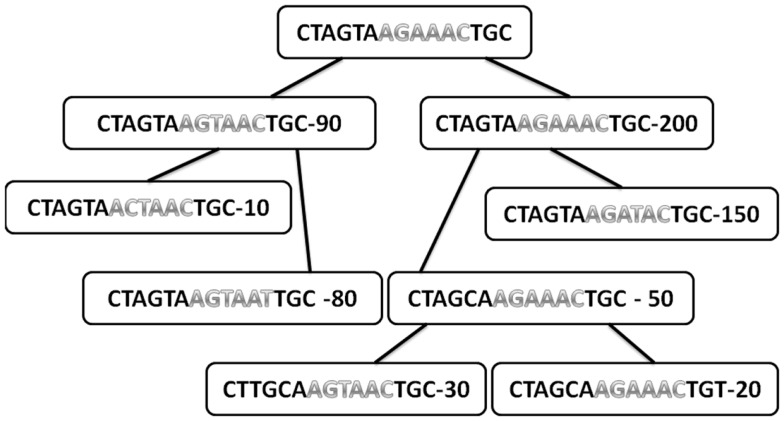
**Typical tree with the sequences in each node and the total number of offspring of each node**. Only internal nodes are drawn here (leaves are not used for the analysis, since they have no mutations under them). The sequences are divided into two regions (different colors). The number in each node is the total number of offspring.

Given a tree, each mutation event is assigned: (a) a NS or S mutation flag by its effect on the amino-acid translation of the containing codon; (b) the location of the mutation (related gene where applicable, and number of nucleotides from the beginning of the sequence, otherwise); and (c) the log of the ratio between the number of leaves (sequences) in the sub-tree following the mutation branch and the number of leaves in the sub-tree following the non-mutated branch (see Figure 1; Figure A1 in Appendix). This ratio is denoted the Log Offspring Number Ratio (LONR). This log-ratio is thus positive if the number of final sequences marked by the tree construction algorithm as descendants of the mutated sequence is larger than the number of final sequences marked as descendants of the non-mutated sequence. This suggests some better fitness of the mutated sequence, or positive selection for such mutation, and negative in the opposite case. For each area of the sequence, a *t*-test is performed (unpaired, unequal variances) between the NS and S mutations (see Figure A4 in Appendix for flow chart).

### Simulation

A sequence pool simulating neutral reproduction was generated from a random original sequence of 348 nt, with a constant multiplication rate of two offspring per organism. Two regions were defined with uniform mutation probabilities with average mutation rate of 1/2 and 1 mutation per generation. The population was sampled in different sample sizes and along different generations. In each sampling, one of the first eight siblings (the third generation) was chosen randomly, and its descendants had a twice higher probability of being sampled, effectively simulating sampling bias for a specific clone. The process was repeated 1000 times, and selection was computed in the described process. NS and S mutations were defined relative to their direct ancestor, resulting in unequal NS and S probabilities. All mutations had similar probabilities (i.e., we did not differentiate between Purines and Pyrimidines).

### Statistical analysis

For the immunoglobulin data, the receptors where clustered by isotype (IgA and IgG). Lineage trees were constructed and the sequences were divided into CDR and FWR regions. Mean LONR NS-S difference was computed per clone and per region along with two sample *t*-test *p*-values.

### Immunoglobulin sequences

Over 500,000 BCRs were sampled from each donor in 12 donors (17), using 454 sequencing, and a RACE protocol. The details of the sequencing and the validity checks are beyond the scope of this manuscript. For each sequence, the most fitting VH, JH, and V-J distance was found by maximizing the relative number of non-mutations for both VH and JH segments. Only sequences that matched higher than 0.5 in both segments were kept for further analysis. The sequences were then clustered according to the most fitting VH and JH as well as the distance between VH and JH, and were truncated to 159 nt from the end of the germline VH and 20 nt from the beginning of the germline JH. Only trees with a sufficient number of mutations were analyzed, which automatically removed sequences defined with ORF and pseudo VH genes (Figure A3 in Appendix).

### Clone definition

Sequences were grouped into clones using a two-step approach. First, the germline VH and JH of each sequence were determined by aligning all possible germline VH and JH (based on the IMGT germline library) (18) against the sequence using the Basic Local Alignment Search Tool (BLAST) (19).

Next, in order to count the clones, we grouped all sequences according to their VH and JH usage as well as the distance between VH and JH, since SHMs usually do not produce additions or deletions of nucleotides. Thus, every clone emerging from the same founder cell should have the same distance between VH and JH. We then took all of the sequences with the same VH, JH, and distance between VH and JH and grouped them using a phylogenic approach. The distance between VH and JH was computed by positioning the IMGT germline VH and JH genes on the observed sequence and determining the distance between the last nucleotide of VH and the first nucleotide of JH.

All the sequences with equal VH, JH, and distance were aligned together with an artificial sequence composed of the germline and gaps between them. Within each group, the sequences were aligned (using MUSCLE 3.6) (20), and a phylogenetic tree was built using maximum parsimony (21) and/or neighbor joining (22) methods (from the PHYLIP 3.69 program package). We then parsed this tree with a cutoff distance of four mutations into clones. Thus, a clone was defined as a set of sequences that are similar one to each other, up to a distance of four mutations.

## Results

### Selection

Before discussing B cells specifically, let us discuss how selection can be estimated in a rapidly mutating population. Assume a population originating from a single founder through asexual division. In the case of B cells, this would be a clone seeded by an ancestral B cell with a given H chain rearrangement. We ignore the L chain at this stage. The genetic sequence of the founder can be changed by mutations that can affect the population dynamics. In such a case we would define positive selection in the population as an increased average division/birth rate or a decreased average death rate following mutations. Note that these are not precisely the same, especially in the context of B cell dynamics (23), but this is beyond the scope of the current analysis. A decrease in the division rate would be defined as negative selection. Note that each mutation by itself can have a positive, null, or negative effect, but the definition of selection is based on the average population dynamics and not with the dynamics following a single mutation.

Let us follow a mutation that occurs within a population. If this mutation increases the average number of offspring per generation from μ to μ + Δμ, then by a time proportional to log of the total population size, the advantageous mutation will take over the population. When we compare the population to its latest common ancestor (LCA), we will have no evidence that such a mutation has occurred (Figures 1A,B). If the original sequence cannot be known (as often occurs in the CDR3 region), we will not be able to detect the presence of such a mutation. If the original sequence is known (as typically occurs in mutations within the germline VH gene), the genetic composition of the population would be equivalent to the one expected in a neutral model (model with no selection). The only difference would be the addition of a single NS mutation to a gene in the entire population. This information can be used to infer that selection has taken place. This is basically the logic behind S to NS mutation ratio tests for selection.

During the intermediate period when the two sub-clones still exist (the mutated and the un-mutated one), one can compare the population size of the two sub-clones. We expect the ratio between the two population sizes to be proportional to *e*^Δμ^*^T^*, where *T* is the time from the mutation to the sampling time (Figure 1A).

For a single mutation, it will be hard to differentiate between the effect of selection and a non-uniform sampling where one branch is sampled more deeply. However, if many mutations occur in the genetic region of interest, and if on average mutations in this region increase the average number of offspring, we expect more offspring in branches that follow a mutation in this region than in branches emerging from the same direct ancestor with no mutations, and inversely in the case of negative selection.

We thus propose to detect selection using this imbalance in cases where the total mutation rate (mutation rate per organism multiplied by population sample size) is significantly higher than one, as typically occur in within clone B cell evolution.

### Log offspring number ratio

We define a measure of selection in a gene as the ratio of the number of leaves (measured descendants) under the branch where a mutation occurred and the number of decedents in its direct sibling where no such mutation occurred. We compare the distribution of these ratios (more precisely the log of the ratios) in S and NS mutations to estimate whether the distribution deviates from the one induced by neutral drift (Figure 1D).

**Figure A2:**
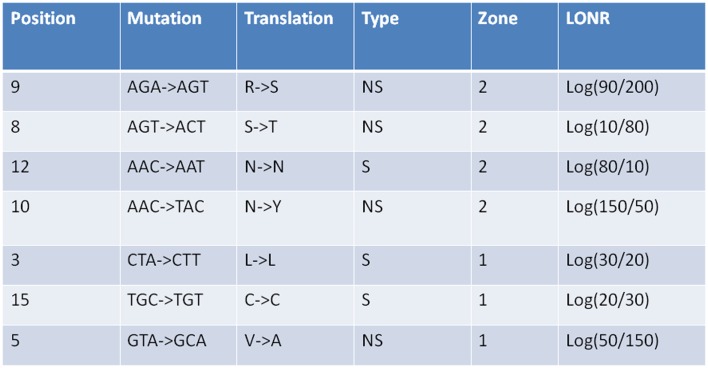
**Log offspring number ratio values for S and NS mutations as given in Figure A1**. For each mutation its region is give (1 or 2), its type (S or NS), its position along the gene in nucleotides, the mutation itself in nucleotides, and amino acids, as well as the LONR score. The distribution of LONR scores is used to assess selection.

**Figure A3:**
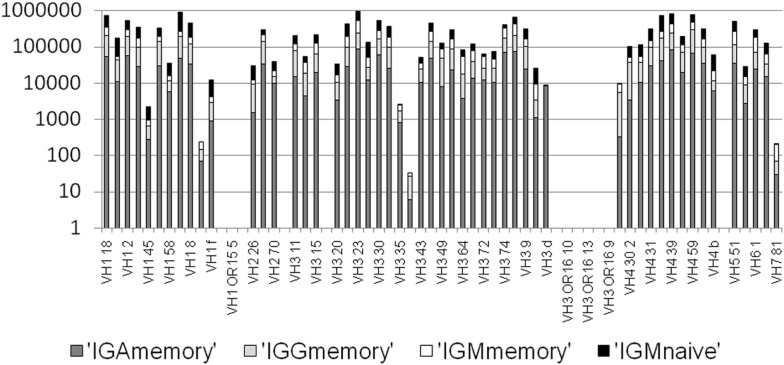
**Number of mutation events per VH gene per isotype**. There are practically no mutations assigned to pseudogenes, since practically none of the sequenced in-frame lineage trees are based on pseudogenes (<0.05%). However, there are also some functional VH genes with practically no lineage trees. We removed those from the current analysis.

**Figure A4:**
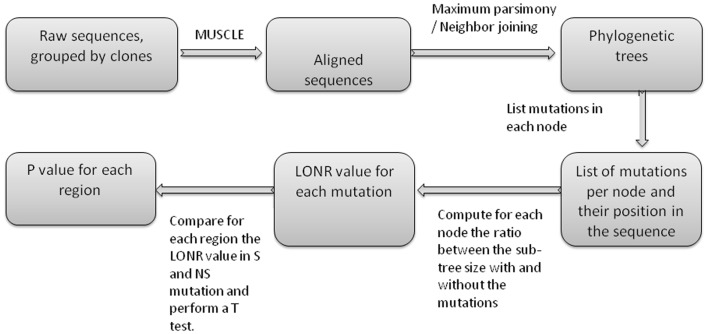
**Flow chart of LONR score production**. First sequences are aligned, then phylogenetic trees are produced. For each internal node in the trees, a list of mutations is produced (between the internal node and one of its direct descendents). For each such mutation the LONR score is computed resulting in a list of LONR score for each mutation. For genetic region, the LONR score of the mutations in this regions are analyzed and the resulting *p*-value is the result of a *t*-test between S and NS mutations.

Specifically, for each mutation occurring in one son of an internal node and not in the other, we compute the sub-tree size under the son with a mutation and the sub-tree under the son without a mutation. The log of the ratio between these two sizes is defined as the LONR of this mutation. We then compute the LONR value for all S and NS mutations in the tree, and compare the S and NS LONR distributions (Figure 1C; Figures A1 and A2 in Appendix).

Note that this analysis is not sensitive to the details of the baseline model for the probability of either silent or replacement mutations, since their absolute number is never used in the analysis. The only case where such a model would affect the current measurement is in the extreme case that the probability for S would differ by orders of magnitude from the probability of NS mutations.

In order to check that the LONR does not detect selection in its absence, we simulated mutating clones, sampled the resulting sequences (see Materials and Methods for details), produced lineage trees, and compared the LONR distribution following S and NS mutations. When the number of mutations is very small, or the number of samples is small, the False Positive (FP) rate (the cases where the LONR average is significantly different following S and NS mutations with a *p*-value of 0.05) is higher than the expected 5%. However, in the regime of over 10–20 mutations per sequence and at least 300 sequences per tree, the FP rates are near the expected 5% (data not shown). We have repeated the analysis with non-uniform mutation rates (position dependent mutation rates) and with sampling biases, and obtained similar results, as long as the S and NS mutation rates are of the same order of magnitude. A detailed methodological analysis of the LONR will be given in a separate analysis.

### Selection along human Ig clones

We have used the LONR score to analyze the healthy repertoire from 12 donors. In such a repertoire two opposite forces operate: (a) mutations can ruin the functionality of the receptor and decrease its survival probability, (b) mutations can on the other hand increase the affinity to the antigen and thus lead to a higher division rate. The CDRs of the BCR determine its interaction with the antigen, and mutations there were reported to have a higher probability to increase the affinity than mutations in the framework (FWR) region (5, 24). However, the net selection effect in each of these regions still remains unclear. Beyond the effect of SHM, B cells are affected by isotype switches from naïve IgM to memory IgM, and from there to memory IgG and IgA. The memory (IgM, IgG, and IgA) isotypes occur at the advanced stages of the immune response and thus lineage trees based on such receptors are expected to represent the full evolution following selection.

We have used high-throughput sequencing to sequence over 500,000 BCR samples from each donor, in 12 donors. We built lineage trees from the sequences [see (17) for details of sequences, and production of lineage trees]. We measured the LONR distribution in all naïve and memory, IgM as well as IgA and IgG sequences trees (over 50,000 lineage trees) and compared the LONR distribution in NS and S mutations. The results are actually quite striking. We analyzed separately CDR2, FWR3, and CDR3, using the standard IMGT definition (18).

We did not analyze the CDR1 and FWR1 and FWR2 regions, since we did not have enough samples with reliable sequences in these regions. Thus, our results only apply to the comparison of the more 3′ regions (CD2,3 and FWR3). Also, we here include the JH region within the CDR3 analysis. This was done in order to avoid artifacts of the DH gene length. In this specific point, our notation slightly deviates from the standard IMGT analysis that ends the CDR3 region at the beginning of the JH gene region.

**Figure 2:**
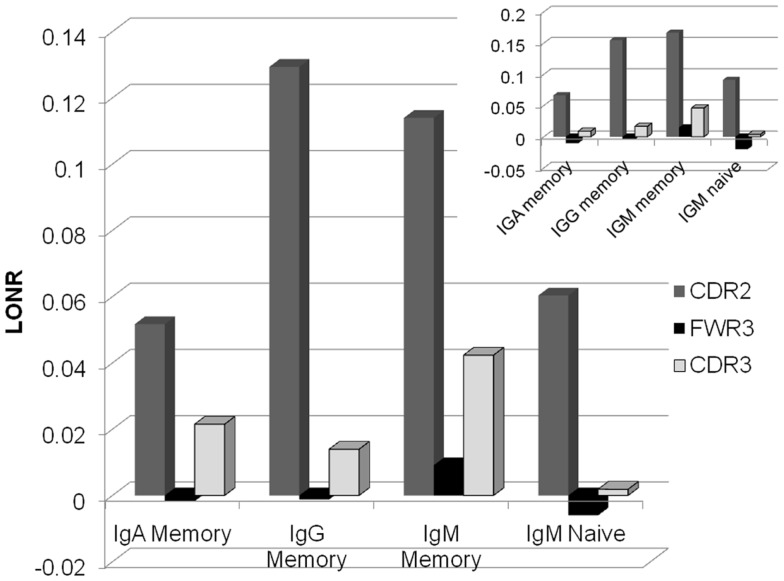
**Average LONR score per region and per isotype for all lineage trees (main figure) and for trees with a significant difference (*t*-test, *p* < 0.01; inset)**. The main positive selection occurs in CDR2, followed by CDR3. FWR3 has a limited if any negative selection. Selection occurs mainly in the memory isotype, but some systematic selection is already observed in the IgM naïve isotype.

As expected in both IgG and IgA memory cells, the positive selection is much stronger for the CDR regions than for the FWR (Figure 2). However, in the memory IgM, even the FWR region passes a positive selection during the immune response. Thus, one cannot conclude as a generic conclusion that FWR regions are under negative selection, while CDR regions are under a positive selection. Within different CDR regions, CDR2 is under a much more stringent positive selection than the CDR3 region. Highlighting the fact that while the CDR3 is selected at clone level, where clones with an appropriate CDR3 sequence are selected for expansion, mutations in the CDR2 region may induce a much stronger positive selection than mutations in the CDR3 that can induce a more balanced effect. When analyzing only trees where there is a significant difference between the LONR score (*p* < 0.01) of S and NS mutations, the results are qualitatively similar to the analysis of all trees (Figure 2, inset).

In order to ensure that the LONR is not an artifact of the sampling depth or the number of mutations, we computed a correlation between the average S vs. NS mutations LONR difference in each sample (a sample being defined as a single donor, a given isotype, and a given VH gene) and the sample size, or the average number of mutations in each region separately or in all regions in this sample. In all cases there was practically no correlation (the highest Spearman correlation was *R* = 0.05). The same occurs if S or NS mutations are used separately. Thus, the observed selection effect is not a sampling or a mutation rate effect.

### Comparison of isotypes

Selection and mutations are accompanied by an isotype switch process. It is not clear from the current literature whether isotype switch precedes mutations and selection or if it occurs in parallel. If selection occurs only following isotype switch, we expect no selection to be observed in lineage trees composed of purely IgM sequences. However, the selection level in IgM memory trees is as high as the one observed in IgG lineage trees, and higher than the one observed in IgA lineage trees. Moreover, even in trees composed only of naïve (CD27−) sequences (25, 26), a clear advantage in the division rate following mutations in the CDR2 region is observed. One can thus conclude that even before they become memory cells, B cells pass an antigen induced selection. Note that the CD27− cells can be activated cells, and are not of a pure naïve type (27, 28).Thus, the observed mutations and selection may actually represent an activated phenotype which is a part of the CD27− sub population.

### Comparison between donors and between VH genes

**Figure 3:**
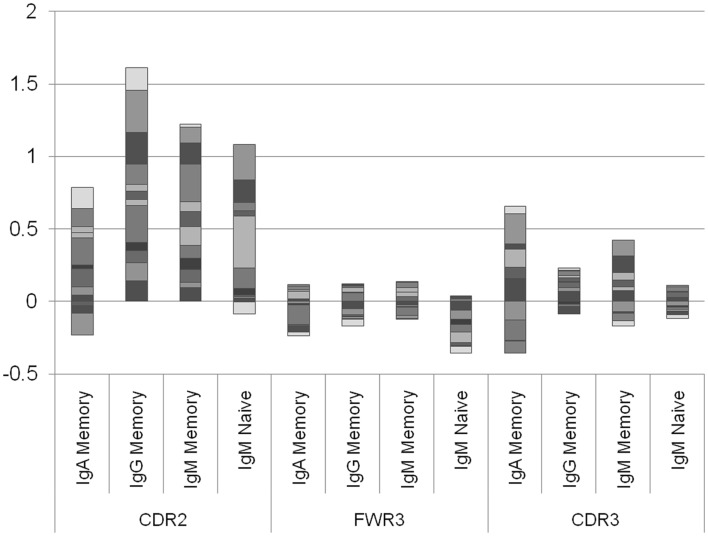
**Log offspring number ratio per donor and per isotype**. Each column is an isotype in a different region, and each color is a different donor. Each column represents the aggregate over many donors, where negative and positive values are drawn separately. While in the CDR2 there are practically no donors where the average selection is negative (with the exception of two IgA samples and one naïve IgM sample), in the CDR3 there are approximately the same number of donors with negative selection and with positive selection. This shows that the selection in CDR2 is a universal feature, while in CDR3 it may depend on the random junction initially produced or in the different exposures to antigens.

The results presented in Figure 2 are the average of the selection score over many donors. Some variability exists between donors, and the average selection score can represent a combination of positive selection in some donors and negative selection in others. We have thus separated the analysis into different donors (Figure 3). A clear difference emerges between the different regions. While in the CDR2 region practically all donors show a positive selection in all isotypes, in FWR3 and CDR3, the results are highly variables in all isotypes, with some donors showing a marked positive selection and some a negative selection. The main difference between FWR3 and CDR3 is not in the sign of the selection but in its variance. In CDR3 the variance among donors is much larger than in FWR3.

### Position effect

**Figure 4:**
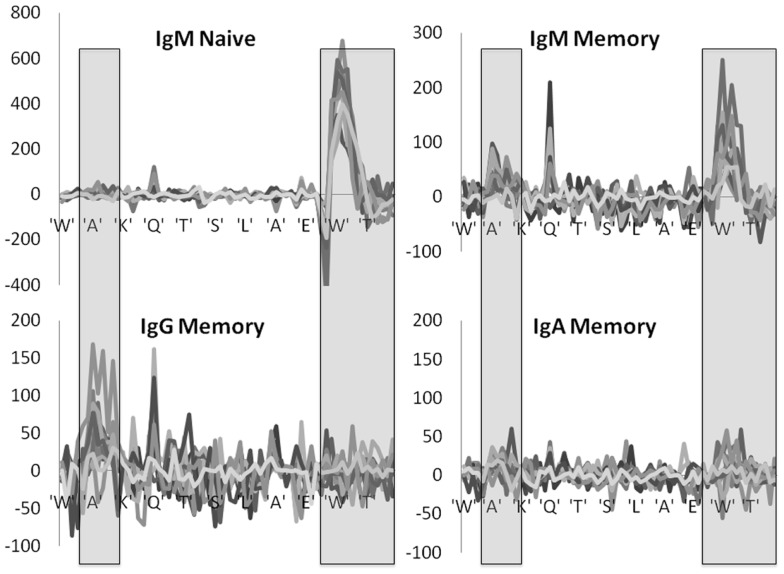
**Position specific effects**. Total LONR score per amino acid (averaged over the 3-nt composing the amino acid). The LONR is drawn for the four isotypes discussed in previous figures: naïve IgM, memory IgM, IgG, and IgA. Each line represents a different donor. The highlighted regions represent the CDR2 (to the left) and CDR3 (to the right). The sequence at the bottom is a typical sequence. The zone of very strong negative selection at the beginning of the CDR3 is the junction region. One can observe a switch from a very strong positive selection in the naïve IgM isotype focused on the CDR3 to a much weaker selection in the IgG and IgA focused on the CDR2. Note that the scales of the y axes are different between the plots.

A more complex picture emerges when each position is analyzed by itself, instead of merging all positions belonging to the same region. At the naïve IgM level, positive selection is mainly focused on the DH region within the CDR3 region, while negative selection takes place in the junction regions (Figure 4). When moving to the IgM memory isotype the selection in the CDR3 becomes much weaker, and the selection in the CDR2 starts to rise. Finally, when moving to the IgA and IgG isotype selection is fully focused on CDR2. Note that we here plot the net selection per nucleotide, without considering the total number of mutations per nucleotide. Thus some nucleotide may contribute significantly more than other nucleotides to the average. This different weighting induces some quantitative differences between Figures 3 and 4. Note also that while the total number of mutations per sequences is much larger in IgG and IgA than in the naïve IgM serotype, the selection is actually maximal at the naïve level.

These results are highly consistent among the different independent donors, with selection patterns practically overlapping in 10 donors out of 12 (Figure 4). In two donors the observed selection was weak and noisy, but these donors had much smaller sample sizes (data not shown).

These results suggest the following two stage selection process occurring in Ig lineage trees. The first stage of selection occurs early in the CDR3 and is generic and uniform. This would be equivalent to the “key mutation” concept (29, 30). Following these key mutations, selection becomes much weaker and focused on the CDR2 regions in the IgG isotype. The memory IgM shows a translational mutation distribution from the key-mutation selection event, to the weaker mutation in the CDR2, which may alters the affinity in a limited way.

**Figure 5:**
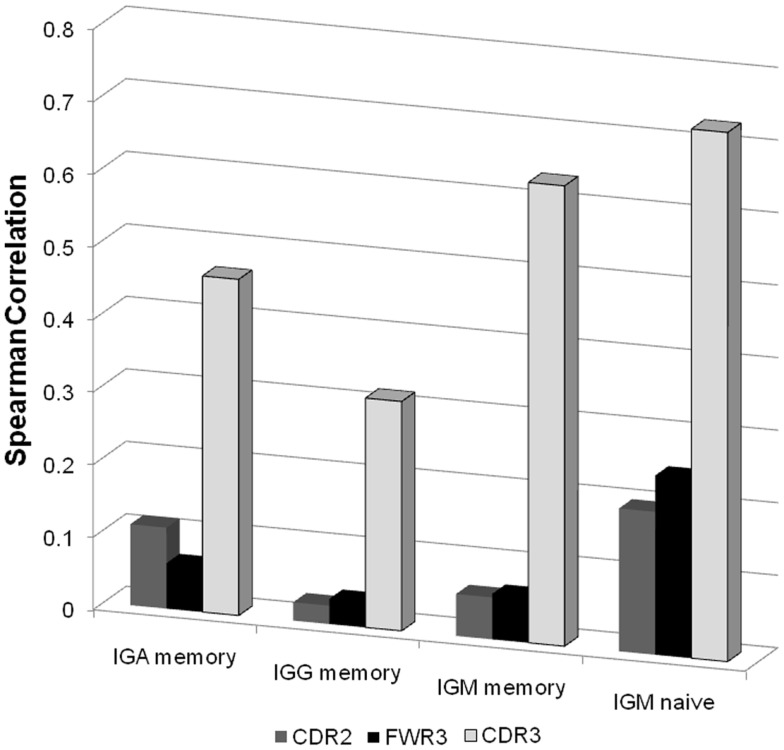
**Spearman correlation between the NS/(S + NS) fraction and the total number of B cells for different isotype and different regions**. For each sample we produced a vector of 46 values (all functional VH genes with enough samples) representing the total number of B cells in this sample with such a V gene. When all samples are taken together, this leads to a 12*2*46 values (12 donors, 2 technical repeats per donor). We computed the correlation of these values with the NS/(S + NS) values in the same categories. As one can clearly see the highest correlation is with the CDR3 regions of naïve IgM samples.

In order to check that there is indeed a correlation between mutations in the CDR3 regions of naïve cells, we compared the correlation between the NS/(S + NS) ratio and the total number of B cells for different regions, VH genes, and isotypes (Figure 5). The total clone size was defined as the number of sequences with a give VH in a give sample. The NS and S mutations were defined as the average number of mutations in the leaves compared with the ancestral sequence of each clone (a similar analysis with the number of unique mutations led to similar results). The total number of B cells is the total number of B cells sequenced in a given sample with the same VH gene. As expected the highest correlation was with the NS/(S + NS) ratio in the CDR3 region of naïve IgM cells. Note that a trivial correlation is expected between the number of B cells and the total number of mutations, since large clones may be older clones and as such accumulate a large number of mutations. However, here we have compares the NS fraction, which is not expected to be correlated with the population size, unless selection is involved.

## Discussion

While multiple sequence based methods have been proposed to detect selection (10, 31–36), most of them are sensitive to the baseline mutation model, or to sampling effects. Moreover, many existing methods conclude the existence of positive or negative selection from highly frequent or rare mutations. Such an under or over expression of a sequence can simply represent the random expansion of a population, a bottleneck effect, or the random association of this mutation with another mutation which is selected. Indeed even in models of neutral evolution, alleles carrying some sequences are expected to be much more frequent than others, since alleles, and sequence distributions have a fat tail. Instead, positive selection should be defined by the systematic increase of the population size following replacement mutations in a given region.

We have here used this precise definition to define the LONR score as the increase/decrease in the relative branch size following a mutation. A comparison between the LONR score following synonymous and NS mutations. A detailed methodological analysis is left to a different framework; we here describe an application of the LONR score to immunoglobulin clones in healthy volunteers.

The LONR differs in two basic aspects from most other S to NS mutation frequencies methods. First this method does not count the absolute number of sequences; instead it measures for each mutation the effect it has on its number of offspring. The second difference is the definition of mutations and their classification as S/NS in respect to their direct ancestor, and not to a consensus or ancestral sequence.

Since each mutation is counted once, independent of the total number of sequences that end up containing this mutation, it is practically not affected by sampling biases or by the expansion of specific sub-populations. Moreover the LONR does not require a baseline mutation model, since it does not compare the number of synonymous or NS mutations. Instead, it measures the effect of each mutation on the total number of offsprings.

Tree shape based methods were developed (11–14). However, these methods often cannot detect the direction of selection, and cannot detect which region in the sequence is selected. Moreover, many of these tree shapes are sensitive to sampling effects making them impractical to use in realistic situations (37).

The observed selection in Ig clones has a well conserved pattern among donors. It is consistently positive in the CDR2 region, and positive in average in the CDR3 region. The mutation pattern in the CDR3 region is composed of strong positive selection in the DH region, and strong negative selection in the junctions between the VH and DH genes and between DH and JH genes. We currently have no clear explanation for the negative selection in the junction. However, one can hypothesize that only B cells with appropriate junctions are selected to pass affinity maturation, and that following within clone selection must maintain these junctions. Such a behavior has been clearly observed in H chain transgenic models of selection in mice (23). At later stages, selection is focused on the CDR2 region and is much lower in the CDR3 regions. This can represent a fine tuning of the affinity, where the main limiting step is the accumulation of key mutations in the CDR3, which is then followed by the expression of more specific mutations in the CDR2 region.

We did not analyze the CDR1 and FWR1 and FWR2 regions, since we did not have enough samples with reliable sequences in these regions. We can only guess that CDR1 should behave approximately like CDR2. FWR1 and FWR2 may be quite different than FWR3 as previously proposed (38). Advances in B cell sequencing technologies (39) will hopefully provide longer reads allowing us to study these regions as well.

## Conflict of Interest Statement

The authors declare that the research was conducted in the absence of any commercial or financial relationships that could be construed as a potential conflict of interest.
